# Rho GTPases in mammalian spinal neural tube closure

**DOI:** 10.1080/21541248.2016.1235388

**Published:** 2016-10-21

**Authors:** Ana Rolo, Sarah Escuin, Nicholas D. E. Greene, Andrew J. Copp

**Affiliations:** Newlife Birth Defects Research Centre, UCL Great Ormond Street Institute of Child Health, London, UK

**Keywords:** actomyosin; Cdc42; cell protrusions; morphogenesis; mouse; neurulation; Rac1; RhoA

## Abstract

Neural tube closure is an important morphogenetic event that involves dramatic reshaping of both neural and non-neural tissues. Rho GTPases are key cytoskeletal regulators involved in cell motility and in several developmental processes, and are thus expected to play pivotal roles in neurulation. Here, we discuss 2 recent studies that shed light on the roles of distinct Rho GTPases in different tissues during neurulation. RhoA plays an essential role in regulating actomyosin dynamics in the neural epithelium of the elevating neural folds, while Rac1 is required for the formation of cell protrusions in the non-neural surface ectoderm during neural fold fusion.

## Neural tube closure

The vertebrate neural tube is the embryonic anlage of the entire central nervous system (brain and spinal cord). It forms from an initially flat neural plate, and undergoes dramatic morphogenetic movements during early development that bend it and shape it into a hollow tube covered by non-neural tissue. Neural tube closure, or primary neurulation, is complete by embryonic day 10 in mouse and by Carnegie stage 12 in humans (approximately 26 d post ovulation). Failure in completion of neural tube closure leads to clinically important birth defects such as anencephaly or open spina bifida, depending on whether the cranial or caudal regions of the tube remain open, respectively.^1^

**Figure 1.**
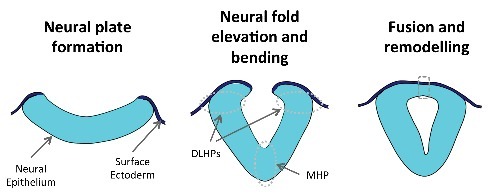
Schematic representation of key steps during mammalian neurulation. The neural epithelium and the surface ectoderm initially form a continuous layer (left and middle panels). As development proceeds, the neural folds elevate and the neural epithelium bends at discrete hinge points (middle panel; MHP – median hinge point and DLHPs – dorsolateral hinge points). The final steps of neurulation involve epithelial fusion and tissue remodeling at the dorsal midline (dashed box, right panel), originating a closed neural tube covered by surface ectoderm.

The neural plate originates as a thickening of the dorsal region of the outer embryonic layer (ectoderm), and is initially continuous with the non-neural surface ectoderm (SE). Gradually, the neural folds (lateral edges of the neural plate) elevate and bend inwards, eventually becoming apposed at the dorsal midline, where they will fuse together. After tissue remodelling, the neural epithelium (NE) and the SE are no longer continuous with each other, but rather form *de novo* adhesions with the concurring tissue on the contralateral side, thus originating an internal closed hollow neural tube covered by SE (Fig. 1).

Mammalian neural tube closure initiates at embryonic day 8.5 at the brain/spinal cord boundary (closure 1), and proceeds bi-directionally in both rostral and caudal directions. In mouse there are 2 additional closure initiation sites in the brain region, whereas the spinal region closes by zippering from closure 1 toward the caudal end of the embryo.

Unlike the amphibian neural plate, which bends evenly along its apical surface, the amniote neural plate bends at discrete sites: the medial hinge point (MHP), at the ventral midline, and 2 paired dorso-lateral hinge points (DLHPs) on either side of the neural plate (Fig. 1). This bending pattern is regulated by mutually antagonistic signals from tissues ventral and dorsal to the neural tube, mainly SHH from the underlying notochord and BMP2 from the SE.^2,3^

### Rac1, Cdc42 and RhoA

Rho GTPases are a family of highly conserved Ras-related small GTPases. Their best studied members, Rho, Rac, and Cdc42, are present in all eukaryotic life forms.^4^ They function as molecular switches that are active when bound to GTP and inactive when bound to GDP. Their activation is promoted by guanine-exchange factors (GEFs) that stimulate exchange of GDP for GTP, and their inactivation is induced by GTPase-activating proteins (GAPs) that catalyze hydrolysis of GTP to GDP.

RhoA, Rac1, and Cdc42 were first identified as key regulators of the cytoskeleton in the early 1990s in a series of papers by Hall and colleagues. Rac1 and Cdc42 induce the formation of cell protrusions, with Rac1 inducing the formation of lamellipodia and membrane ruffles, and Cdc42 inducing filopodia.^5,6^ RhoA, on the other hand, promotes actomyosin contraction and formation of stress fibers.^5,7^ As expected from molecules involved in cell motility and cytoskeleton regulation, these small GTPases have since been shown to be involved in many aspects of embryonic development and morphogenesis.^8-10^ In particular, RhoA/ROCK signaling is required for convergent extension movements at the onset of mouse neural tube closure,^11,12^ but further roles for Rho GTPases in the subsequent steps of neurulation were not firmly established until recently.

This mini-review focuses on the roles of Rho GTPases in mouse spinal neural tube closure, particularly in light of 2 recent papers from our group analyzing the roles of RhoA and actin dynamics in the NE^13^ and Rac1 and membrane protrusions in the SE.^14^

### RhoA and actomyosin dynamics during bending of the spinal neural plate

**Figure 2.**
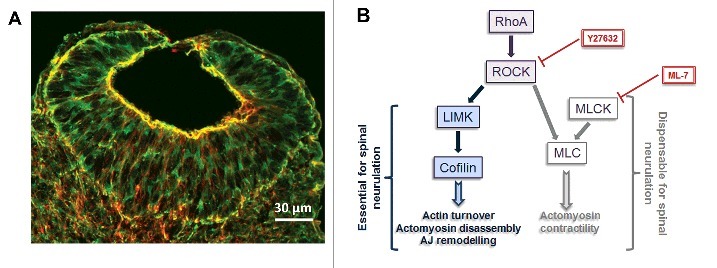
RhoA/ROCK and actomyosin regulation in the NE. (A) Phalloidin (actin, red) and myosin II (anti-MHCB, green) immunostained transverse section through the recently closed region of the spinal neural tube of a E9.5 mouse embryo showing apical accumulation of actomyosin (yellow) in the NE cells. (B) Summary of RhoA/ROCK signaling requirements during spinal neurulation. RhoA/ROCK signaling upstream of LIMK/Cofilin is required for spinal neural tube closure by regulating cytoskeletal and adherens junction (AJ) dynamics, and these processes can be inhibited by the RhoA/ROCK inhibitor Y27632, whereas actomyosin contractility downstream of RhoA/ROCK/MLC is dispensable for neurulation, which is not inhibited by the MLCK inhibitor ML-7. Adapted from ref. 13.

Apical actomyosin-driven constriction is a widely used mechanism to shape and bend epithelia, such as in sea urchin and *Drosophila* gastrulation or in *Xenopus* bottle-cell formation.^15,16^ There is marked apical accumulation of actomyosin in NE cells (Fig. 2A), and prevailing theories postulate that apical constriction driven by actomyosin contraction is responsible for neural plate bending. This seems in fact to be the case for mammalian cranial neural tube closure, which can be inhibited in vitro by actin disassembling drugs such as cytochalasins.^17-19^ Furthermore, several mouse mutants for genes encoding cytoskeletal proteins exhibit exencephaly.^20^ The vast majority of these mutants nevertheless successfully close their caudal neural tubes, and the same is true for cytochalasin-treated embryos, raising the idea that actomyosin contractility is not essential for spinal neurulation. In addition, actomyosin does not accumulate focally at the NE bending points, but rather is present at the apical ends of cells along the entire dorso-ventral extent of the neural plate (Fig. 2A). Other lines of evidence indicate that cell wedging at neuroepithelial hinge points might not result from active apical constriction. MHP cells in mouse, like in chick, become wedge shaped as a result of prolonged cell cycle, which leads to basal nuclear localization in the pseudo-stratified NE. The discrete bending at DLHPs results from buckling of the NE at a dorso-ventral point in which there is a change in cellular density.^21^

The recent work in our group by Escuin and colleagues^13^ has shed new light on the roles of RhoA and actomyosin dynamics in mouse spinal neurulation. They confirmed that cytochalasin and other actin polymerisation inhibitors, at concentrations just below the toxic limit, have no effect on spinal neural tube closure. The same was true for Blebbistatin, an inhibitor of actomyosin crosslinking, notwithstanding the fact that all these drugs perturb apical actomyosin localization. On the other hand, drugs (Y27632 and HA-100) that inhibit RhoA's target Rho kinase (ROCK) and which, via negative feedback, also inhibit RhoA itself,^22^ were shown to affect spinal closure. This effect was concomitant with a dramatic increase in apical actomyosin accumulation and an expansion of the adherens junction (AJ) domain (seen by an enrichment of apical β-catenin/F-actin clusters). Interestingly, Blebbistatin was able to rescue the effects of Y27632 treatment, both restoring spinal neural closure and normalizing the distribution of actomyosin and AJ components, suggesting that the neurulation defects caused by RhoA/ROCK inhibition result from abnormal apical F-actin accumulation. In fact, treatment with jasplakinolide, a drug which inhibits actin depolymerisation, caused the same effects as RhoA/ROCK inhibition, and could equally be rescued by co-treatment with Blebbistatin.

ROCK is responsible for the phosphorylation of targets LIMK and myosin light chain (MLC). Phosphorylated MLC (pMLC) is essential for myosin II contractility, and MLC can be phosphorylated by either ROCK or MLCK. ML-7 is an MLCK inhibitor, and Escuin *et al.* showed that in mouse embryo cultures it effectively reduces the levels of pMLC, without affecting the levels of phosphorylation of either LIMK or its target cofilin. ML-7 treatment also reduced the apical levels of actomyosin, without, however, affecting spinal neurulation. Conversely, analysis of cofilin-null mouse embryos showed that these have delayed spinal closure, together with an increase in apical actomyosin accumulation and AJ components, similar to that seen in RhoA/ROCK-inhibited embryos (and similarly rescuable by Blebbistatin treatment).

Escuin's work thus not only confirms early studies indicating that actomyosin contractility is dispensable for mammalian spinal neurulation, but also shows that fine-tuning of actomyosin levels with a particular requirement for regulated actin depolymerisation is critical for closure. This precise regulation of actomyosin dynamics is regulated by a signaling cascade involving RhoA/ROCK/LIMK/Cofilin (Fig. 2B). When RhoA/ROCK signaling is inhibited, actomyosin disassembly and turnover through this pathway becomes misregulated, whereas MLC can still be phosphorylated by MLCK, leading to an overall increase of apical actomyosin. This effect can be rescued by simultaneously inhibiting actomyosin (as in treatment with Blebbistatin).

It is important to note that in the inhibited embryos analyzed by Escuin and colleagues, DLHPs formed despite the neurulation defects observed, indicating that these defects may result from increased tension in the NE, rather than from a bending defect. Mouse embryos treated with cytochalasin have ‘floppier’ neural folds, which nonetheless achieve closure,^23^ whereas increased stiffness (as you'd expect in embryos with increased apical actomyosin) likely makes them mechanically opposed to closure. Hence, in mouse spinal neural tube closure it appears that actomyosin-related NE tension is a ‘passive’ requirement for closure, which needs to be maintained within strict limits in order to enable closure to proceed. The factors that ‘drive’ neural tube closure remain to be determined but likely include changes in cell density, dorso-ventrally, within the NE which may underlie the propensity of the neural folds to bend at DLHPs, leading to apposition of the fold tips and fusion.

### Rac1 and Cdc 42 regulate membrane protrusions during neural fold apposition

**Figure 3.**
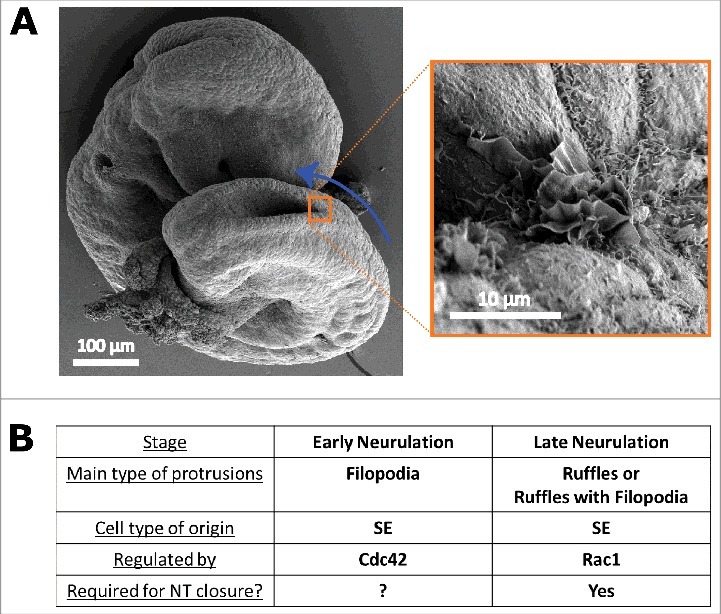
Roles of Rac1 and Cdc42 in protrusive activity in the SE. (A) Scanning electron micrographs of an E9.5 mouse embryo showing membrane ruffles at the site of spinal neural fold apposition. Blue arrow indicates the direction of zippering of the spinal neural tube. Right sided image is an enlargement of the orange boxed region. (B) Summary of the roles, regulation, and requirement of different types of protrusions during spinal neurulation, as described in ref. 14. Rac1 is essential for the formation of membrane ruffles in SE cells at late neurulation, and their absence results in neural tube defects, whereas Cdc42 regulates formation of filopodia by SE cells during early neurulation.

Cell protrusions are present at the edges of elevated mammalian neural folds, before they become apposed at the future dorsal midline^24,25^ (Fig. 3A). As described above (and in Fig. 1), at this stage the NE is continuous with the SE, and the tips of the neural folds correspond to the border between these 2 tissue types. In the mid- and hindbrain regions, the initial contact between contralateral folds is established by SE cells, and it is from these same cells that protrusions emanate, whereas in the forebrain contact is initiated by NE cells.^24,26,27^ The cell type of origin of the protrusions seen in the spinal neural folds was previously unknown. Our recent work ^14^ has looked at the origin and regulation of protrusions during spinal closure, and, importantly, at their requirement for completion of neural tube closure.

Using scanning electron microscopy to characterize protrusion morphology in detail, we showed that in the early stages of spinal neurulation there are predominantly filopodia at the tips of the neural folds. Gradually, as neurulation progresses along the spinal axis, these become replaced by either a mixed type of protrusions, with both ruffles and filopodia (the latter often emanating from a lamellipodial veil), or by membrane ruffles alone, devoid of filopodia. To target these protrusions, address the question of which tissue type produces them, and whether they are essential for neurulation, we used a genetic approach in which we crossed conditional (floxed) knock-out mice for either *Cdc42* or *Rac1* with Cre lines driving recombination in the NE and/or SE.

Knocking-out *Rac1* in the SE leads to open spina bifida, with the timing of onset of the defect (and, consequently, the size of the lesion) correlating with the stage at which recombination of *Rac1* occurs in the SE cells. Concomitantly, there is a shift in the type of protrusions seen in these embryos, with either a trend toward the production of filopodia instead of membrane ruffles or, in the most extreme case, with a complete absence of protrusions. Ablation of *Rac1* in the NE, however, does not lead to neurulation defects or changes in protrusive activity. Interestingly, unlike RhoA/ROCK-inhibited embryos,^13^ *Rac1* conditional mutants do not display AJ defects in either the SE or the NE, despite Rac1 being known to regulate the formation of AJs in other systems.^28-30^

Unlike *Rac1*, when *Cdc42* is knocked-out in the SE at late neurulation stages it does not cause neurulation or cell protrusion defects. When *Cdc42* is knocked-out from the SE earlier in development, however, there is a shift in the type of protrusions observed, and instead of the filopodia typical of early neurulation, these embryos display membrane ruffles instead. Because this early removal of Cdc42 from the SE caused lethality before completion of neurulation, it is thus impossible to determine whether this change in protrusive activity would result in neural tube defects.

In summary, our recent work shows that cell protrusions are required for successful completion of spinal neural tube closure, and that the type of protrusions changes with time. In early neurulation stages they are mainly filopodial and are regulated by Cdc42, whereas in late neurulation they are predominantly membrane ruffles (either alone or mixed with filopodia) and are regulated by Rac1 (Fig. 3B).

While our work was the first to provide evidence that protrusions are indeed required for neurulation, the question remains of how protrusions act to promote neural tube closure. Even though in other epithelial closure events (such as *Drosophila* dorsal closure, for example) cell protrusions are thought to mechanically exert traction to bring tissues together,^31^ this does not seem to be the case in neural tube closure. Firstly, the protrusions emanate into a fluid-filled space, rather than crawling on an extracellular matrix or cellular substrate where they could exert traction and, secondly, even in the absence of protrusions the neural folds still elevate and DLHPs form. It is thus conceivable that the protrusions instead serve as exploratory structures that upon recognition of other protrusions during fold apposition initiate a series of events leading to the tissue rearrangements and remodelling necessary for completion of neurulation.

The epithelial fusion and remodelling events of neurulation remain largely uncharacterised and are likely to involve further roles for small GTPases. In the recently closed SE, immediately rostral to the fold apposition point, no cell protrusions are detected. This cessation of protrusive activity in the cells upon remodelling of the SE into a continuous epithelium might be akin to formation of *de novo* cell adhesions in MDCK cells, in which after initial contact by Rac1-driven lamellipodia, E-cadherin accumulates and the initial contact spreads by RhoA-induced actomyosin contraction, while simultaneously Rac1 and lamellipodia are inhibited.^30^ It is also possible that Rac1 and Cdc42 are involved again later in neurulation completion, during tissue remodelling, particularly in re-establishment of apico-basal polarity and epithelial adhesions of the SE or the NE. In *Drosophila* dorsal closure, the leading edge cells undergo an incomplete epithelial-to-mesenchymal transition caused by loss of apico-basal polarity.^32,33^ As the cells meet in the midline and zipper along the gap, they restore their polarity and cell-cell adhesions, and these events are dependent on Pak, a well-known target of Rac1 and Cdc42.^32^ In fact, in our work we show that Cdc42 ablation in the NE, despite not causing neurulation defects, does lead to disrupted neural tube organization after closure, likely resulting from a requirement of Cdc42 for neuroepithelial polarity.^34^

### Rho GEFs and GAPs in mouse neurulation

Given the roles for RhoA and Rac1 in mouse neurulation described above, it is expected that Rho GTPase activators and repressors (GEFs and GAPs, respectively) may also be key players. In other vertebrate models, specific Rho GEFs have been shown to be required for neurulation. *Xenopus* morphants for GEF-H1 show neural tube defects (NTDs) and disrupted MLC phosphorylation,^35^ and in chick embryos PDZ-RhoGEF co-localizes with myosin cables at the apical end of NE cells, with its depletion resulting in NTDs and loss of apical myosin cables.^36^ However, KO mice for *PDZ-RhoGEF* neurulate normally and are viable.^37^ In fact, most Rho GEF mutant mice generated to date are viable, and all successfully complete neurulation,^38^ except *ect2* mutants, which die before implantation.^39^ This is perhaps expected, given the high degree of redundancy of Rho GEFs, which can be appreciated by the fact that there are 4 times as many GEFs as there are GTPases.^40^ This number is even more extreme for RhoA, Rac1 and Cdc42, which have at least 25 different GEFs each.^38,41^ The only GAP known to be essential for mouse neurulation is p190 RhoGAP, with mutant mice for this gene showing exencephaly with a penetrance of 30%, together with increased basal F-actin accumulation in floor plate cells.^42^

### Different roles and tissue requirements for Rho GTPases in spinal neurulation

**Figure 4.**
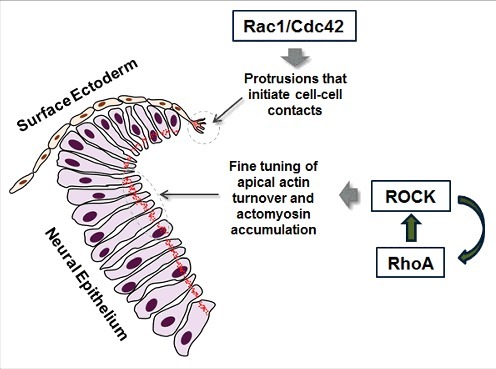
Rac1/Cdc42 and RhoA are both required for neural tube closure. Schematic representation of the roles of Rho GTPases during spinal neurulation. Rac1/Cdc42 are required in the SE for the formation of protrusions that initiate contacts with contralateral SE cells. RhoA is essential in the NE, where it regulates the balance of assembly and disassembly of actomyosin. F-actin is depicted in red.

Overall, our recent papers^13,14^ show that both Rac1 and RhoA play key roles in spinal neural tube closure in addition to the initial function of RhoA in neural plate convergent extension. These GTPases have, however, not only different functions, but are also required in different tissue types (Fig. 4). Rac1 (and possibly Cdc42) is required in the SE for the formation of membrane protrusions that initiate contact with the contralateral side. RhoA and its effector ROCK, on the other hand, are required in the NE and are responsible for maintaining balanced apical actomyosin accumulation and regulating actin turnover.
